# Evolution and Expression Patterns of *CYC/TB1* Genes in *Anacyclus*: Phylogenetic Insights for Floral Symmetry Genes in Asteraceae

**DOI:** 10.3389/fpls.2017.00589

**Published:** 2017-04-25

**Authors:** María A. Bello, Pilar Cubas, Inés Álvarez, Guillermo Sanjuanbenito, Javier Fuertes-Aguilar

**Affiliations:** ^1^Plant Evolutionary Biology Group, Real Jardín Botánico (CSIC)Madrid, Spain; ^2^Department of Plant Molecular Genetics, Centro Nacional de Biotecnología, CSIC-Universidad Autónoma de MadridMadrid, Spain

**Keywords:** *Anacyclus*, Asteraceae, *CYC2* diversification, *CYCLOIDEA*, *CYC/TB1*, floral symmetry

## Abstract

Homologs of the *CYC/TB1* gene family have been independently recruited many times across the eudicots to control aspects of floral symmetry The family Asteraceae exhibits the largest known diversification in this gene paralog family accompanied by a parallel morphological floral richness in its specialized head-like inflorescence. In Asteraceae, whether or not *CYC/TB1* gene floral symmetry function is preserved along organismic and gene lineages is unknown. In this study, we used phylogenetic, structural and expression analyses focused on the highly derived genus *Anacyclus* (tribe Anthemidae) to address this question. Phylogenetic reconstruction recovered eight main gene lineages present in Asteraceae: two from *CYC1*, four from *CYC2* and two from *CYC3*-like genes. The species phylogeny was recovered in most of the gene lineages, allowing the delimitation of orthologous sets of *CYC/TB1* genes in Asteraceae. Quantitative real-time PCR analysis indicated that in *Anacyclus* three of the four isolated *CYC2* genes are more highly expressed in ray flowers. The expression of the four *AcCYC2* genes overlaps in several organs including the ligule of ray flowers, as well as in anthers and ovules throughout development.

## Introduction

Asteraceae is the largest family of vascular plants with more than 23,600 species distributed among 13 different lineages (Panero et al., 2014). By contrast, its closest relatives, Calyceraceae and Goodeniaceae, have around 54 and 404 species, respectively (Carolin, 2007; Pozner et al., 2012). The evolutionary success of Asteraceae is strongly associated with its head-like inflorescence or capitulum (Broholm et al., 2014). Variations in perianth morphology, symmetry and sexuality of the flowers along the radial axis of this inflorescence have been used to discriminate different types of capitula (i.e., bilabiate, ligulate, radiate, discoid, and disciform; Jeffrey, 1977; Bremer, 1994). This diversity has important consequences, such as differential attractiveness to pollinators, increased rate of outcrossing and fitness by the presence of peripheral ray flowers (e.g., *Senecio*; Chapman and Abbott, 2009), or differential rates of seed germination across the capitulum (e.g., *Anacyclus*; Torices et al., 2013).

Floral symmetry is one of the most striking morphological variations within the capitulum. The evolution of floral symmetry in angiosperms is controlled by a restricted set of gene families (Luo et al., 1996; Costa et al., 2005; Broholm et al., 2008; Hileman, 2014). Among these, the TCP factor *CYCLOIDEA* (*CYC*) (Luo et al., 1996) has been recruited for independent transitions but then further modified or lost in conjuction with reversals to radial symmetry. The TCP gene family (Cubas et al., 1999) has expanded 600–800 million years ago, forming the class I and class II TCP subfamilies (Navaud et al., 2007). The TCP class II subfamily is formed by the *CYCLOIDEA/TEOSINTE BRANCHED1* (*CYC*/*TB1*) and *CINCINNATA* (*CIN*) clades (Martín-Trillo and Cubas, 2010). The *CYC/TB1* or ECE clade is specific to angiosperms and underwent duplication before the core eudicot diversification, producing three main groups: *CYC1, CYC2*, and *CYC3* (Howarth and Donoghue, 2006; Citerne et al., 2013). *CYC3*, and particularly *CYC1* genes, have a major role in the regulation of axillary bud outgrowth (Aguilar-Martinez et al., 2007; Finlayson, 2007), whereas *CYC2* genes control the growth patterns of flower meristems and regulate the establishment of bilateral symmetry of flowers in asterids and rosids (Busch and Zachgo, 2009; Preston et al., 2011; Nicolas and Cubas, 2015).

Investigation of Asteraceae *CYC/TB1* gene family evolution performed in *Gerbera* (Mutisieae), *Helianthus* (Heliantheae), and *Senecio* (Senecioneae) revealed that *CYC2* genes play a critical role in controlling ray flower identity (Broholm et al., 2008, 2014; Chapman et al., 2008; Kim et al., 2008; Juntheikki-Palovaara et al., 2014), and changes in temporal and spatial expression of *CYC2* genes are associated with modifications of the floral symmetry pattern (Busch and Zachgo, 2007; Gao et al., 2008; Zhou et al., 2008). The acquisition of bilateral flowers in Asteraceae involved parallel recruitment of the diversified *CYC2* genes, according to recent phylogenetic reconstruction of *CYC/TB1* genes (Chapman et al., 2008, 2012; Tähtiharju et al., 2012).

We characterized the *CYC/TB1* genes of the Mediterranean *Anacyclus* (Anthemideae), inferred their phylogenetic relationships and expression patterns, and compared them with the identified models in *Gerbera, Helianthus* and *Senecio* in order to test if the bilateral symmetry evolved early in the family due to cooption of CYC2 clade genes. *Anacyclus* L. with homogamous (i.e., all flowers bisexual, tubular and pentamerous flowers) and heterogamous capitula (with modified female, bilateral and trimerous ray flowers surrounding the bisexual, tubular and pentamerous disc flowers) (Bello et al., 2013) is a suitable model with which to carry out this comparative analysis because it is mostly annual, diploid (2n = 18, *x* = 9; Ehrendorfer et al., 1977; Humphries, 1981) and is suitable for *ex situ* cultivation. Also, the floral development of *Anacyclus, Gerbera* and *Helianthus* is similar, excepting the delayed development of the ray flowers relative to the disc flowers in *Anacyclus*, the exclusive presence of transitional flowers in *Gerbera*, the sterile condition of the ray flowers in *Helianthus* and the late zygomorphy of the disc flowers in *Anacyclus* (Laitinen et al., 2006; Tähtiharju et al., 2012; Bello et al., 2013). Moreover, unusual heterogamous capitula with peripheral “trumpet” flowers in natural populations of *A. clavatus* (Desf.) Pers. and *A. valentinus* L. documented from southern Spain (Bello et al., 2013) are very similar to *tub* (Berti et al., 2005) and *turf* (Chapman et al., 2012) mutant individuals of *Helianthus*. These “trumpet” flowers differ from the typical ray flowers in their tubular five-lobed perianth with radial symmetry and the labile presence of stamens (Bello et al., 2013).

As *Anacyclus* represents the derived and highly diversified tribe Anthemideae, where at least 20 genera have species presenting inflorescences with and without ray flowers, we extended the phylogenetic range of the *CYC/TB1* studies. Although there are previous partial Asteraceae *CYC/TB1* phylogenetic reconstructions (Chapman et al., 2008, 2012; Kim et al., 2008; Tähtiharju et al., 2012), a framework to visualize the entire diversification scenario in the family based on nucleotide variation is lacking. With the inclusion of several eudicot *CYC/TB1* sequences in our analysis, together with those available from Asteraceae and the isolated CYC-like genes from *Anacyclus*, we have reconstructed a wider lineage profile and propose it as a model system for the classification and identification of paralogous and orthologous groups of *CYC/TB1* genes in Asteraceae. Having this phylogenetic framework, we have explored if *CYC/TB1* diversification involved positive selection, differential rates of evolution or differential expression patterns of the paralogs in Anacyclus.

## Materials and methods

### Plant materials

Seeds and entire plants of wild *Anacyclus clavatus* (IA 2006, Soto del Real, Madrid), *A. valentinus* (LM 4435, Altea, Valencia), *Matricaria aurea* (IA 1995, El Retiro, Madrid) and *Matricaria chamomilla* (IA 1996, living collection Real Jardín Botánico, Madrid) were collected in 2008–2009 and treated as indicated in Bello et al. (2013). *A. clavatus* with trumpet phenotypes were obtained from seeds collected in May 2012 (one population from Carchuna, Granada), sowed in November 2010 and harvested in May 2013.

### *Cycloidea* gene analysis

*CYC*-like genes of *Anacyclus* and *Matricaria* species were amplified from genomic DNA and cDNA with previously published (Chapman et al., 2008) and own-designed primers (Table S1). The DNeasy® and RNeasy® Plant Mini kits from Qiagen® were used for DNA and RNA extraction, respectively. RNA extraction of individual plant tissues was performed after their dissection, fixation and disruption in liquid nitrogen. RNA concentration was measured by spectrophotometry (NanoDrop 1000 v3.7, Thermo Fisher Scientific Inc.) and adjusted among tissues. cDNA synthesis was performed with the Invitrogen™ ThermoScript™ RT-PCR system kit. Semi quantitative RT-PCR of CYC-like amplicons from young roots (10-cm-long), leaves (2-cm-long), peduncules (1-cm-long), capitula (ca. 1 cm diameter), inflorescence bracts (ca. 0.5-cm-long), young/full expanded ray flowers (1–2.5-cm-long) and young/mature disc flowers (0.5–1.5-cm-long) of *A. clavatus* was carried out three times using different CYC and actin primer sets (Table S1). PCR amplification was performed with Ready-To-Go PCR beads (Illustra™) using a general program as follows: 95°C/5 min followed by 35 cycles of 95°C/30 s, annealing temperature for 30 s and 72°C/45 s, and a final extension of 72°C/7 min (annealing temperatures are listed in Table S1). Amplified sequences were cloned using the Promega pGEM®-T Easy vector system (JM109 competent cells) and sequenced on a 3730 DNA Analyzer (Center for Research Support CAI, Universidad Complutense, Madrid) and an ABI 3700 instrument (STAB VIDA DNA sequencing service, Oeiras, Portugal). Inverse PCR and RACE techniques were used to amplify longer sequences from *Anacyclus*. For inverse PCR, DNA from *A. clavatus* and *A. valentinus* was extracted (Doyle and Doyle, 1987) and digested with 1.5 μL of restriction enzyme (*Bam*HI, *Pst*I, *Eco*RI, *Hin*dIII, *Xho*I, *Nco*I; New England Biolabs® Inc.) in a reaction containing 1 μL of DNA, 2 μL of buffer, and 15.5 μL water. After 3 h of incubation (37°C) and 10 min of enzyme deactivation (65°C), the reactions were diluted (280 μL water) and ligation was performed using 26 μL of DNA, 3 μL of ligase buffer and 1 μL of ligase. Ligation products were amplified with specific primer pairs (Table S2) and cloned for sequencing. For 3′ RACE amplification of *CYC* genes from young capitula of *A. clavatus*, the SMARTer™ RACE cDNA Amplification Kit and Advantage® 2 PCR Taq polymerase (Clontech Laboratories, Inc.) were used with the specific primer Ha2c_11 (Table S1).

### Phylogenetic analysis

Selected clones isolated from *Anacylus* (79) and *Matricaria* (16) were aligned with other *CYC*-like genes from other species of Asteraceae (44) and other eudicots (54) including Calyceraceae and Goodeniaceae (Table S3). Initial alignments were performed with Geneious Pro 5.5.5 (Biomatters, http://www.geneious.com/; Kearse et al., 2012) using the default options of Geneious, MUSCLE and ClustalW. Nucleotides were aligned considering the codon arrangement in the amino acid alignment. Nucleotide (4) and amino acid (1) matrices were assembled (Table S4). The models of evolution for CYC amino acid (JTT + I + G, −lnL = 11,683.44) and nucleotide matrices (GTT + I + G, −lnL = 22,128.4089) were estimated using ProtTest 3 (Darriba et al., 2011), jModelTest 2 (Darriba et al., 2012) and Modeltest V 3.8 (Posada and Crandall, 1998). For Bayesian analyses (Ronquist et al., 2012), the matrices were analyzed with Mr Bayes 3.2.2 on XSEDE as implemented in the CIPRES Science Gateway (http://www.phylo.org/sub_sections/portal; Miller et al., 2010). For the amino acid matrix, the model was set to fixed (Jones), the rates to gamma distribution, the number of generations to 5,000,000, the number of chains to 1, and the sample frequency to 2,000. The nucleotide analyses were performed with and without removal of the third codon. In two other data sets, ambiguously aligned positions were removed and *CYC2* genes from Asterales were analyzed independently (Table S4). For these analyses, the number of chains was set to four with 15,000,000 generations, a sample frequency of 2,000 and a diagnostic frequency of 5,000. The selected outgroup for all analyses was *AcTBLb* (*Acorus calamus*) except the *CYC2* genes from Asterales dataset that used *SlCYC1* (*Solanum lycopersicum*) as outgroup. In all cases, the post-burn in trees were selected after discarding 25% of the trees. Final toplologies were visualized with figTree v1.1.2 (http://tree.bio.ed.ac.uk/software/figtree). Mapping of the CYC amino acids along the trees was done with Mesquite 3.0 (Maddison and Maddison, 2011) using parsimony optimization. The amino acids were tracked on a post burn-in tree (tree 4,800,000) resulting from the amino acid Bayesian analysis. Maximum likelihood (ML) analysis was performed with GARLI 2.1 (Bazinet et al., 2014) and the bootstrap support was estimated with a 1,000 replicate search in Bootstrap RAxML (Stamatakis et al., 2008) in the CIPRES portal.

### Diversifying/purifying selection of CYC genes

Recombination Detection Program RDP v4.36 (Heath et al., 2006) was used to identify potential cases of recombination that could affect the estimate for selection, implementing the RDP (Martin and Rybicki, 2000) and MaxChi (Smith, 1992) methods. To detect individual sites subject to episodic diversifying selection, the CYC nucleotide matrix was analyzed under the mixed effects model of evolution (MEME) and the fixed effects likelihood approaches (FEL) in Datamonkey (Delport et al., 2010). In MEME, the distribution of the rate ω varies from site to site and also from branch to branch in a site, capturing the footprints of episodic and pervasive positive selection, whereas in FEL the synonymous and non-synonymous rates are fitted at each site with no variation along branches (Kosakovsky Pond et al., 2005; Murrel et al., 2012).

### Diversification rates analysis

We used BAMM (Rabosky et al., 2014) to estimate rates of diversification across different gene lineages across the CYC phylogeny. The general model in this Bayesian method assumes that phylogenetic trees may have been shaped by a heterogeneous mixture of different evolutionary rates of gene diversification and extinction. Our working hypothesis was that, given a balanced sampling across paralogs of CYC2 in Asteraceae, which exhibit a number of paralogs larger than in other eudicot families, we could detect a significant heterogeneity across branches of arising paralogs in Anthemideae. We allowed each regime to be characterized by a distinct time-varying speciation process, where the diversification rate varies exponentially through time. The model of exponential change has been used in taxon diversification studies and is also expected as an approximation to diversity-dependent changes in gene diversification rates through time (Rabosky, 2014). We accounted for incomplete taxon sampling using the analytical correction implemented in BAMM, assuming that our sampling included 95% of extant *Anacyclus* CYC diversity. Visualization was performed using R scripts available through the R package BAMMTOOLS (Rabosky, 2014; Rabosky et al., 2014).

### Expression analysis by quantitative PCR

Expression of the *CYC* genes was compared in wild rayed and “trumpet” inflorescences of *A. clavatus* using young plant tissues: roots (10-cm-long), leaves (2-cm-long), capitula stage 1 (≤ 0.5 cm diameter), capitula stage 2 (>1 cm diameter), mature ligules (>3-cm-long, full expanded) and closed disc flowers (0.5-mm-long). Three different individuals of wild and “trumpet” *A. clavatus* were included in the analysis, as well as three technical replicates of each tissue. RNA extraction was performed as described above. cDNA synthesis was carried out with the Transcriptor Universal cDNA Master kit® (Roche) using the following conditions: 25°C/5, 55°C/10, and 85°C/5 min. RNA concentration was adjusted among tissues, adding up to 15 ng/μL per reaction. The qPCR was run on a LightCycler 2.0 using 4 μL of Sensimix Capillary Kit, 0.2 μL of Sybr green and 0.75 μL of MyFi™ DNA Polymerase (Bioline) together with designed lineage-specific CYC primers (final concentration 0.2 μM, Table S5). Positive (genomic DNA) and negative (without nucleic acids) controls were included in each qPCR run to test the resultant crossing-point (Cp) values. Discarded Cp values included those higher than the Cp of the negative control, values above 35, and dissimilar melting temperatures compared against the positive control. Actin was used as the reference gene and was amplified in all tissues. The primer efficiency (E) was calculated from the amplification of three replicates of the “capitula stage 2” tissues, contrasting the logarithm of the fluorescence against the Cp and applying *E* = 10^(1/slope)^ (Pfaffl, 2001; Table S6). E was calculated with the wild and trumpet tissues (Table S5). For the relative quantification, all Cp values were normalized against the Cp from “capitula stage 1” tissue (Cp control) and the E target ^ΔC^ /E control ^ΔC^ ratio calculated (Pfaffl, 2001). For a graphical format of the *CYC* gene expression, the average of three tissue replicates of this ratio was calculated together with the standard deviation. The Kolmogorov-Smirnov test was used to evaluate the probability distribution of the expression average ration in trumpet and wild individuals (α = 0.05, Table S7). A *t*-test paired two sample for means was conducted to test if there is significant difference between the mean expression of AcCYC2 genes in trumpet and wild individuals (α = 0.05, Tables S8, S9).

### RNA *In situ* hybridization

A non-radioactive *in situ* protocol using RNA probes was followed using wild inflorescences of *A. clavatus* at different developmental stages. Capitula of different stages with a maximum of 1 cm of diameter were dissected under a Leica M165FC stereo microscope and fixed in 4% formaldehyde with 0.1% Tween-20 and 0.1% Triton X-100 (Jackson, 1991). Gene-specific probes for the *CYC2* clade genes *2A, 2B, 2C*, and *2D* were amplified using the following general program: 95°C/5 min, 35 cycles with 95°C/30 s, annealing temperatures for 1 min, 72°C/2 min, and a final extension of 72°C/12 min (Table S10). A sense probe was amplified with the 2b set of primers and used in further analyses. Antisense and sense probes were tested with M13 primers in combination with the *CYC2* primers. Probes were cloned into pBluescript II SK and linearized with BamHI before digoxigenin labeling (Coen et al., 1990), which was performed with anti-digoxigenin-AP, Fab fragments, T7 RNA polymerase and deoxynucleoside triphosphates (Roche). Tissue pretreatment, hybridization, washing and antibody staining steps followed Coen et al. (1990) and Fobert et al. (1994). The reaction to visualize the hybridized probes was incubated for 24–48 h at room temperature (~23°C). Sections were mounted with DePeX mounting medium (Serva) and observed and photographed using a Leica DMR microscope with an Olympus DP70 camera. Images were edited and organized in Adobe Photoshop CS4.

## Results

### Phylogenetic reconstruction reveals eight *CYC/TB1* gene lineages in asteraceae

**Figure 1:**
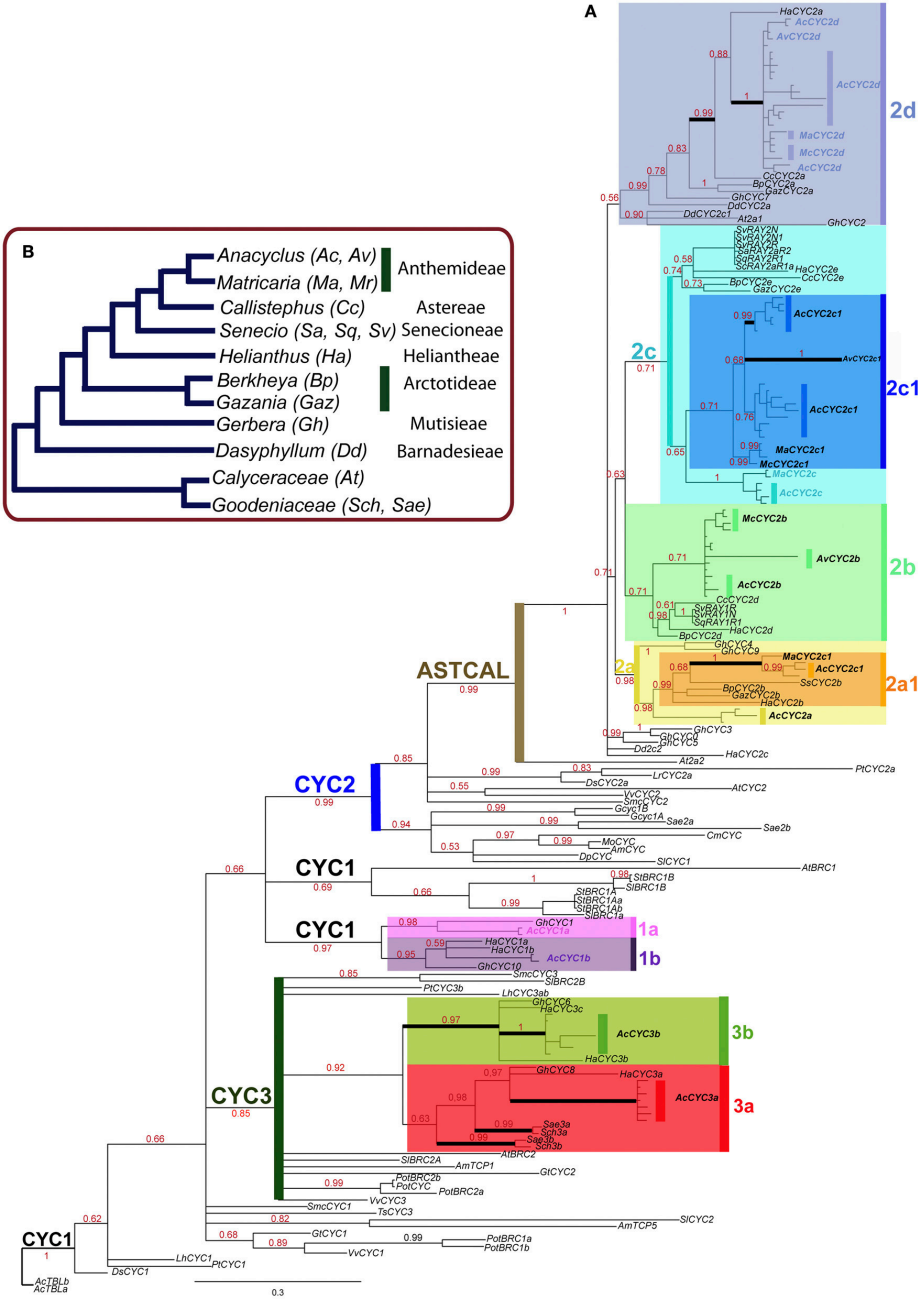
**(A)** Majority rule consensus tree from Bayesian analysis of the nucleotide dataset with excluded third codon positions (tree II) with the main *CYC/TB1* gene lineages identified by colored boxes. Genes from Anthemideae are in bold. Branches affected by episodes of positive selection are indicated by black thick lines. Numbers in red represent posterior probabilities above 0.50. **(B)** Summary tree of the phylogeny of Asteraceae (modified from Panero et al., 2014) illustrating the relationships among the genera included in this study and their corresponding tribes.

The *CYC/TB1* phylogeny reconstruction reveals new and previously identified gene lineages in Asteraceae (Figure 1A). We define a lineage as a group formed by genes from different Asteraceae species representing an orthologous gene set. Although the identified *CYC/TB1* lineages are unevenly sampled for all the Asteraceae species, each of them is congruent with the species phylogeny (Figure 1). For tribe Anthemideae, we identified cDNAs from 10 putative *CYC/TB1* genes (Figure 1) from *A. clavatus* (*AcCYC1a, AcCYC1b, AcCYC2a, AcCYC2a1, AcCYC2b, AcCYC2c, AcCYC2c1, AcCYC2d, AcCYC3a*, and *AcCYC3b*), three from *A. valentinus* (*AvCYC2b, AcCYC2c1*, and *AvCYC2d*), four from *Matricaria aurea* (*MaCYC2a1, MaCYC2c, MaCYC2c1*, and *MACYC2d*), and three from *M. chamomilla* (*McCYC2b, McCYC2c1*, and *McCYC2d*). We also identified several allelic variants for each gene (Table S1, Figure 1). The coding sequences (CDS) range between 783 and 900 bps. In the Bayesian and ML analyses reconstructed from the amino acid and nucleotide data, eight main lineages were recovered (Figure 1A): two formed by “CYC1-type” genes (*CYC1a, CYC1b*), four by *CYC2* genes (*CYC2a, CYC2b, CYC2c, CYC2d*) and two by *CYC3* genes (*CYC3a, CYC3b*). *CYC2a1* and *CYC2c1* are nested lineages in *CYC2a* and *CYC2c*, respectively. *CYC2a1* represents a gene diversification (Figure 1) congruent with the genera phylogeny. The *CYC2c1* genes display diversification of *Anacyclus* and *Matricaria* genes only (Figure 1). The *CYC2a* lineage involves *Gerbera* and *Anacyclus* genes, whereas in *CYC2c*, genes from different species display a diversification congruent with the species phylogeny (Figure 1A). *CYC2b* and *CYC2d* represent relatively well-sampled gene lineages congruent with the species phylogeny.

*CYC2* and *CYC3* genes form monophyletic groups, but their relationship with other clades is unresolved or poorly supported (Figure 1A, Table S11). By contrast, genes formerly identified as “*CYC1*” do not form a single clade. Some “*CYC1*-like” genes, including Solanaceae and the Asteraceae 1A, 1B lineages, are more closely related to *CYC2* genes than to other “*CYC1*-like” eudicot loci (Figure S1, Tree V). In all topologies, the earliest divergent *CYC2* genes are those from Adoxaceae, Brassicaceae, Caprifoliaceae, Gesneriaceae, Goodeniaceae, Plantaginaceae, Solanaceae, and Vitaceae (Figure 1). The remaining *CYC2* members form a well-supported clade with genes from Asteraceae and Calyceraceae (Table S11). Hereafter, this clade will be called the Ast/Cal clade (Figure 1A). A close relationship between *CYC2b* and *CYC2c* is seen in all resultant trees excepting tree V from the GARLI analysis (Figure S1D). All *CYC2* genes from Asteraceae are placed in the CYC2a–CYC2d lineages except some sequences from *Dasyphyllum, Gerbera* and *Helianthus*, which display unstable locations when different topologies are compared (Figure 1, Figure S1, Table S3).

### *CYC/TB1* genes display a pervasive purifying selection with bursts of episodic positive selection

We tested whether the *CYC/TB1* genes included in the phylogeny have been under positive selection. After rejection of recombination in the *CYC/TB1* complete nucleotide matrix by RDP analysis (*P* = 0.05), the dataset was evaluated by MEME and FEL. The analysis with MEME, with higher log-likelihood values than FEL, suggested episodic positive selection for 42 codons (Table S12). The output from FEL indicated negative and neutral evolution for 100 and 146 codons, respectively. Neither MEME nor FEL suggested evidence of pervasive positive selection.

Although most amino acid positions with episodic positive selection within the TCP domain lie in the basic domain and in the loop, additional sites were detected within helix 1 and adjacent to helix 2. The maximum likelihood estimate (MLE) of the synonymous rate α is always higher than the non-synonymous rate β− (β ≤ α) except in a few conservative sites (α = β−) between TCP and ECE domains (Table S12). On the other hand, identified sites with high selective constrain (β− = 0) occur in the TCP domain and outside the ECE and R domains. The proportion of the branches evolving at unconstrained non-synonymous rate β+ is always small (q+ < 38%) in comparison with branches where the synonymous substitutions prevail (q−> 63%). Examination of the magnitude of the Empirical Bayes Factor (EBF) and the single nucleotide substitutions on different branches of the MEME output trees (not shown) revealed that several clades were affected by episodic positive selection (Figure 1). From the identified *CYC/TB1* lineages, the CYC3b clade is the only one affected by episodic positive selection just before its ortholog diversification. Although there is a pervasive purifying selection trend in the *CYC/TB1* genes here analyzed, there are episodes of positive selection not associated with the diversification of the main orthologous gene sets here identified, excepting *CYC3b*.

### Inference of diversification rate shifts

The phylogenetic data coupled with the BAMM model is designed to automatically detect changes of speciation rates. The BAMM analyses converged well as indicated by high ESS values (ESS log-likelihood = 1244.05, ESS number of shifts = 1333.27). BAMM failed to detect any significant rate-shift configuration associated with *CYC* lineage diversification, and the best-fit model to the phylogeny was one involving a homogeneous process of near constant per-lineage diversification rates except for lineage 2c1 restricted to *Anacyclus* (Figure S2). Running the analyses with the different prior settings did not change the overall pattern.

### *AcCYC2b, AcCYC2c*, and *AcCYC2d* genes are highly expressed in wild but not in trumpet ray flowers

**Figure 2:**
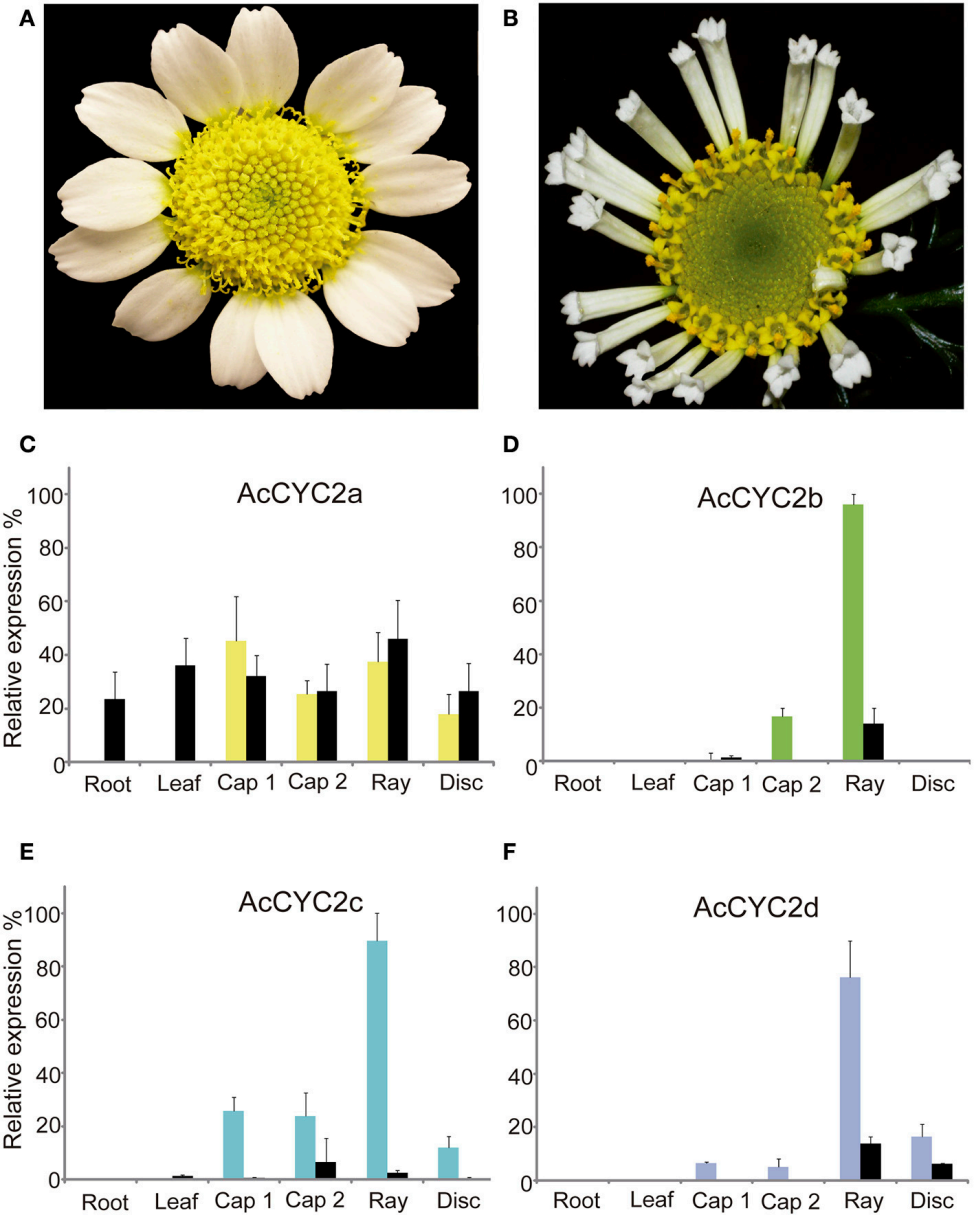
**Inflorescences of wild (A)** and trumpet **(B)** individuals of *Anacyclus clavatus*. **(C–F)** Relative expression levels of *A. clavatus* genes *AcCYC2a*–*AcCYC2d* in vegetative and reproductive tissues of wild (colored bars) and trumpet (black bars) individuals. Bars represent relative differences in gene expression level in the tested tissues and are the average of three biological replicates. Error bars show the standard deviation. Cap 1, capitulum stage 1; Cap 2, capitulum stage 2; Ray, peripheric ray flower (wild type, bilateral) or tubular (“trumpet-type,” actinomorphic); Disc, disc flowers. Photographs by M.A. Bello **(A)** and R. Riina **(B)**.

There was little or no expression of CYC2 genes in vegetative tissues compared with flowers or inflorescences (Figure 2), except for *AcCYC2a* gene expression in roots and leaves of trumpet individuals. In ray and disc flowers, the expression of *AcCYC2a* was not different between wild and trumpet individuals (Figures 2A–C). *AcCYC2a* was expressed at slightly higher levels in young capitula (Cap1) and ray flowers than in mature capitula (Cap2) and disc flowers (Figure 2A). Expression of *AcCYC2a* in wild individuals (not shown) was high (97.45%) in young ray flowers with unexpanded ligules (ca. 0.5-cm-long). In addition, the relative expression of *AcCYC2b, AcCYC2c*, and *AcCYC2d* genes was much higher in ray flowers of the wild individuals than in other tissues (Figures 2D–F). In trumpet individuals, the expression was below 20% in young capitula (Figures 2D–E), mature capitula (Figure 2E), rays (Figure 2D), and disc flowers (Figures 2E–F). The average target EexpΔCt/ control EexpΔCt results for each gene follow a normal distribution according to the Kolmogorov-Smirnov test (α = 0.05, Table S11). The *t*-test indicates that the expression of the *AcCYC2* genes in wild and trumpet is significantly different for all genes (α = 0.05, Table S12).

### Pattern of *AcCYC2* gene expression in early flower development

**Figure 3:**
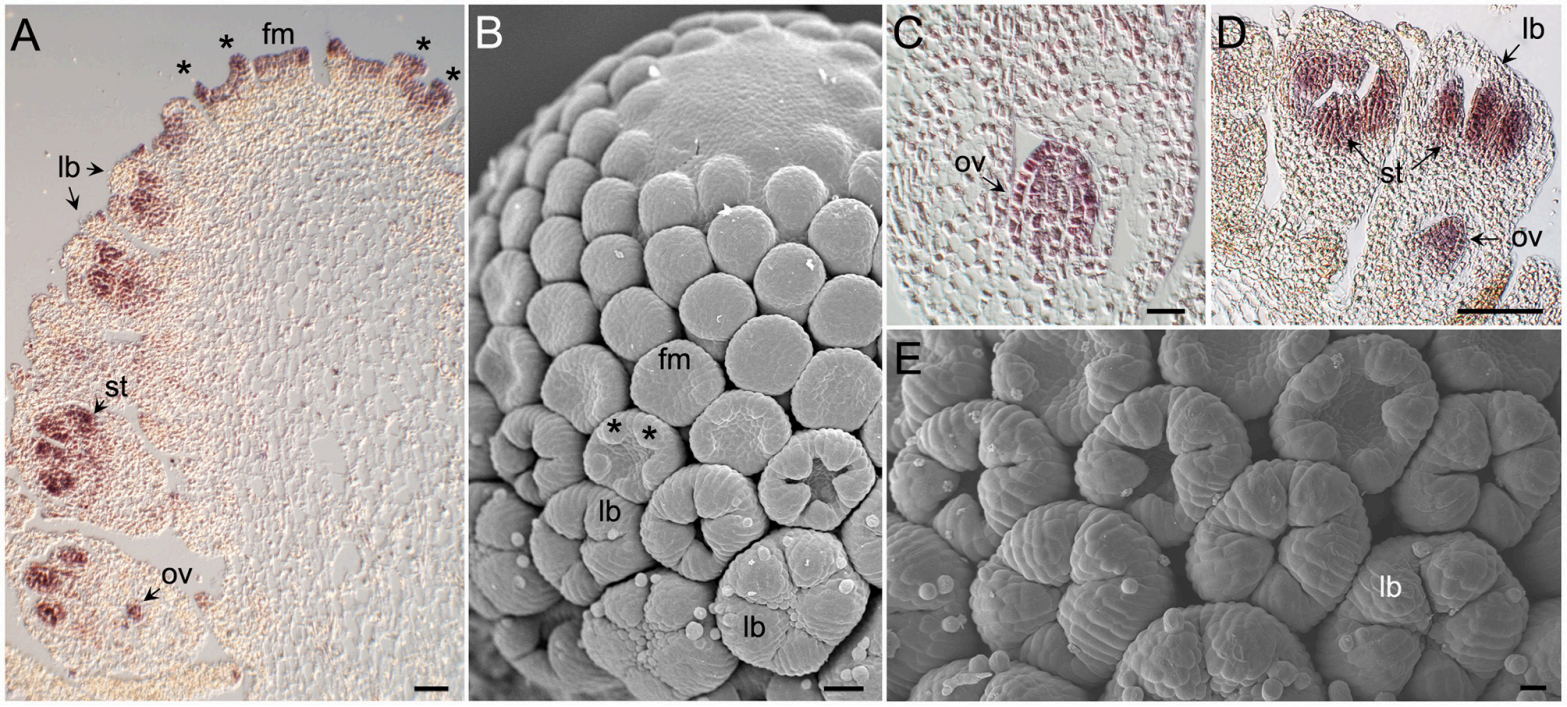
**Morphology of ***Anacyclus*** capitulum and florets, and tissue-specific expression of ***AcCYC2*** genes during capitulum development. (A)** Section of a Anacyclus capitulum hybridized with a digoxigenin-labeled probe complementary to *AcCYC2d*. Youngest flower meristems (fm) can be seen at the top, older flowers to the left and right. *AcCYC2d* transcripts accumulate in young flower meristems and in meristems that are initiating corolla lobe primordia (*). In older flowers mRNA is detectable in developing stamens (st) and ovules (ov). No signal is detectable in developing corolla lobes (lb). **(B)** Scanning electron microscopy (SEM) image of a capitulum of an age comparable to that in **(A)**. **(C)** Close up of an ovule displaying *AcCYC2d* expression. **(D)** Young disc floret showing *AcCYC2a* mRNA signal in stamens and ovule but not in corolla lobes. **(E)** SEM image of disc flowers comparable to those in **(D)**.

To investigate the mRNA distribution of *AcCYC2* genes during capitulum and early flower development we carried out *in situ* hybridizations with digoxigenin labeled RNA probes complementary to these genes. *AcCYC2b* and *AcCYC2c* transcripts could not be detected in our experiments. In contrast, *AcCYC2d* was strongly expressed in young disc flower meristems and during floral organ initiation (Figures 3A,B). *AcCYC2d* mRNA also accumulated in young developing stamens and ovules (Figures 3A,C). Likewise, *AcCYC2a* transcripts were detectable in developing disc flowers, both in the developing stamens and the ovules (Figures 3D,E). At this floral stage *AcCYC2d* and *AcCYC2a* signals were clearly excluded from developing corolla lobes (Figures 3A,D). Sense probes of these genes gave no detectable signal in sections of similar tissues (Figure S3).

## Discussion

### *CYC/TB1* diversification in asteraceae reflects species phylogeny

The phylogenetic reconstruction shown in this study (Figure 1) is congruent with previous partial analyses of *CYC/TB1*-like genes of Asteraceae (Chapman et al., 2008, 2012; Kim et al., 2008; Tähtiharju et al., 2012). The CYC2 orthologous sets proposed by Tähtiharju et al. (2012) correspond to our 2a–2d lineages, with the difference that we found two lineages within 2a (2a1 and 2a2) and a new diversification within Anthemideae (2c1 and 2c2). Within CYC3, we also identified the two orthologous groups 3a and 3b reported by Tähtiharju et al. (2012), and in CYC1 we inferred the lineages 1a and 1b. Although congruence between *CYC-like* genes and species trees is not easy to interpret (e.g., in Dipsacaceae; Carlson et al., 2011), our gene tree reconstruction for each of the *CYC* lineages is broadly consistent with the species tree phylogeny (Panero and Funk, 2008; Figure 1B). For example, in the lineage 2b, the topology of the genes from [(*Berkheya* (*Helianthus* (*Senecio*+*Callistephus*))) (*Anacyclus*+*Matricaria*)] is congruent with the species phylogeny of Asteraceae (Figure 1B). The same consistency is maintained in lineages 1b, 2a1, 2a2, 2b, 2c, and most of 2d, where the only incongruence with the species tree is the closer relationship of *HaCYC2a* from *Helianthus* to *Anthemideae* (*AcCYC2d, AvCYC2d, MaCYC2d*, and *McCYC2d*) rather than to *Callisthephus* (*CcCYC2a*) genes (Figure 1).

Despite this general congruence, the distribution of the *CYC2* genes indicates specific gene gain/loss events in relatively well-sampled genera, such as *Gerbera, Helianthus* and *Senecio*. Tracking the *CYC2* paralogous genes of *Anacyclus* and *Matricaria*, we found that they are distributed in six gene clades, whereas *Gerbera* genes are absent from 2a1, 2b, and 2c clades (however, there is a phylogenetically unstable clade composed of *GhCYC0* and *GhCYC3* with an unresolved position with the CYC2 lineages, which could be potentially included in any of the CYC2 lineages; Figure 1, Figure S1). *Helianthus* genes are absent from the 2a1 clade and *HaCYC2c* relationships are unresolved, while *Senecio RAY1* and *RAY2* are present only in the 2b and 2c clades. Aside from the obvious lack of gene and species sampling that can alter the *CYC/TB1* diversification pattern, it would be important to test whether the differential gene diversification of the species is linked to morphological or functional evolutionary transitions. Moreover, the orthologous *CYC2* genes in *Helianthus* and *Gerbera* do not necessarily share the same function (Broholm et al., 2014), which might allow similar gene repertories shaping different inflorescence morphologies.

The maintenance of each *CYC2* paralog in different species of Asteraceae suggests that they existed before the species diversification had taken place in this family. This recruitment and maintenance of CYC2 function along the history of Asteraceae results in independent evolution of an adaptive trait, the heterogamous capitula (Chapman et al., 2012; Hileman, 2014). The convergent headed inflorescence in Asteraceae and Dipsacaceae could be the consequence of similar diversification patterns of *CYC1*- and *CYC2*-like genes in both families (Howarth and Donoghue, 2005; Carlson et al., 2011; Specht and Howarth, 2014). Although the *CYC/TB1* phylogenetic pattern is not a consequence of differential rate shifts between the inferred CYC lineages according to the BAMM analyses performed here, whole genome duplication events in Asteraceae occurring in the last 40 million years (Barker et al., 2008, 2015) could be responsible for the observed diversity of *CYC/TB1* genes.

Now that several *CYC/TB1* genes isolated from separate studies consistently fit into specific CYC lineages in a comprehensive phylogeny (Figure 1A), it will be convenient from here onto add and assign newly isolated genes to this framework. It will encourage an appropriate association of new *CYC/TB1* isolated genes with its phylogenetic origin and will allow a more consistent classification.

### *CYC/TB1* proteins evolve under purifying and episodic positive selection

Analysis of our *CYC/TB1* gene dataset suggests a pervasive purifying selection with bursts of episodic positive selection, where a very small proportion of sites evolve at unconstrained non-synonymous rate (q^+^ < 38%; Table S12). Chapman et al. (2008) found that the per-site frequency of synonymous substitutions was saturated on many internal branches of *Helianthus CYC/TB1* genes and that TCP and R domain were evolving under strong purifying selection. Similarly, *CYC/TB1* genes from Antirrhineae were also subjected to strong purifying selection (Hileman and Baum, 2003). Even in recent duplicates of *RAY2* in *Senecio vulgaris*, there is no evidence of positive selection that justifies their divergence (Chapman and Abbott, 2009). In terms of events of episodic codon selection associated with the stem/crown clades of the main CYC lineages identified in this study, only one codon has episodic positive selection at the stem node of the lineage CYC3b (Figure 1). For the remaining main CYC lineages, there were no identified episodes of positive selection associated with their origin or diversification, suggesting that selection changes are not important performers of the *CYC/TB1* main phylogenetic patterns. Although some minor clades seem to be diversified after the positive selection (Figure 1), it might be affecting the quaternary rather than the primary/secondary protein structure used for phylogenetic reconstruction. With the lack of a significant difference between the diversification rates in the CYC/TB1 gene lineages suggested by our BAMM analysis, it seems that it is a more uniform and recent pattern of evolution of these genes.

In cases, such as the bird toll-like receptors (Grueber et al., 2014) and the eudicot X-intrinsic proteins (Venkatesh et al., 2015), there is a similar pattern of predominant purifying selection with rounds of episodic positive selection as inferred here for the *CYC/TB1* genes. The high constraints imposed by the purifying selection of *CYC/TB1* proteins could be maintaining their general patterns/functions conserved along the eudicots, whereas the episodic positive selection might allow a subtle modulation of protein-protein interactions, such as binding regulation or protein differential heteromeric combinations (see e.g., differential capacity of dimerization of *CYC/TB1* proteins in *Gerbera* and *Helianthus* in Tähtiharju et al., 2012).

### Expression patterns of *CYC/TB1* are similar in asteraceae orthologs

Comparing the *CYC/TB1* genes of *Anacyclus clavatus* with their identified orthologs, the expression patterns are similar. For example, *AcCYC2b*, detected in young capitula and highly expressed in ray flowers (Figure 2D), lies in the orthologous set of *Senecio RAY1* and *HaCYC2d* (lineage CYC 2b; Figure 1). *RAY1* is expressed in young inflorescences in the peripheral area of the ray floral meristem in radiate and non-radiate capitula of *Senecio* (see Figure 2 in Kim et al., 2008) and *HaCYC2d* is one of the strongest candidates for conferring ray flower identity in *Helianthus* (Tähtiharju et al., 2012). *AcCYC2d* has a similar expression profile to *AcCYC2b* in qPCR analyses (Figures 2F), and is orthologous to *HaCYC2a* and *GhCYC*7 (lineage CYC 2d; Figure 1). *GhCYC7* and *HaCYC2a* are expressed in different tissues, but *GhCYC7* appears in earlier stages of ray and trans flowers, similar to *HaCYC2a* in ray flowers (Chapman et al., 2008; Tähtiharju et al., 2012; Juntheikki-Palovaara et al., 2014). Also, *AcCYC2d* (Figure 3A) and *GhCYC7* are expressed in early stamen primordia of disc and ray flowers (Juntheikki-Palovaara et al., 2014).

Nevertheless, in the lineages CYC2c and CYC2a (Figure 1A, Figure S4), the expression pattern of orthologous genes is not as similar as in *CYC2b* and *CYC2d*. In CYC2c, the orthologous *AcCYC2c, RAY2* and *HaCYC2e* genes are highly expressed in young and mature ray flowers (Kim et al., 2008; Tähtiharju et al., 2012) but *HaCYC2e* appears widely expressed in several tissues in PCR assays (Chapman et al., 2008). In the lineage CYC2a, the genes *AcCYC2a, GhCYC4, GhCYC9*, and *HaCYC2b* are expressed in different tissues, but expression is nonetheless higher in ray flowers of *Gerbera* and *Helianthus* (Figure 3C; Chapman et al., 2008; Tähtiharju et al., 2012). The expression of *AcCYC2a* and *HaCYC2b* seems not affected when actinomorphic tubular ray flowers are formed in the trumpet individual (Figure 2C) and the tubular mutants of *Helianthus*, respectively (see Figure 2B in Chapman et al., 2012). Therefore, despite the fact that in Asteraceae the *CYC2b* and *CYC2d* orthologous genes display similar expression patterns, it is not always possible to predict a particular CYC/*TB1* gene expression pattern from the phylogenetic framework.

In the case of genes with unstable phylogenetic positions, such as *HaCYC2c, GhCYC2, GhCYC3*, and *GhCYC5* lying outside the CYC2 lineages 2a–2d (Figure 1, Figure S1), there are redundant expression patterns and multiple functions (Figure S4). Whereas, *GhCYC5* seems to be involved in the control of the flower density and in floral organ fusion, *GhCYC2* is expressed in the dorsal part of the ray flowers, reproductive whorls, the ligule and the perianth throat of ray flowers (Broholm et al., 2008; Tähtiharju et al., 2012; Juntheikki-Palovaara et al., 2014). *GhCYC3* and *HaCYC2c* are crucial for the ray flower identity and are expressed in meristem, perianth and ovules of ray flowers (Tähtiharju et al., 2012; Chapman et al., 2008). Juntheikki-Palovaara et al. (2014) suggest that redundancy of the *CYC2* genes in *Gerbera* reflect a functional specificity for the CYC2 proteins obtained by the formation of specific protein complexes. In Asteraceae, the maintenance of the inflorescence unit may require a cross regulation between the *CYC2* genes from different lineages, analogous to the interactions of *CYC2* genes identified in *Primulina heterotricha* from Gesneriaceae (Gao et al., 2008; Yang et al., 2012).

A gradient of expression of the *CYC/TB1* genes occurs in pseudanthial structures bearing different morphologies (e.g., in the Myrtacean *Actinodium cunninghamii* where a cluster of fertile actinomorphic flowers are surrounded by ray-shaped branched shoots; Claßen-Bockhoff et al., 2013). This pattern is coincident with the centripetal gradient of floral morphology of the Asteracean inflorescence (Harris, 1999; Citerne et al., 2010). Aside from *HaCYC2b* in wild type *Helianthus* (Chapman et al., 2012), the *CYC2* genes of wild *Anacyclus* (*AcCYC*), *Gerbera* (*GhCYC*), and *Helianthus* (*HaCYC*) are usually highly expressed in the zygomorphic ray flowers relative to the disc flowers (Figure 3; Chapman et al., 2012; Tähtiharju et al., 2012). Expression patterns in *Helianthus* mutants agree with this general Asteraceae profile (Berti et al., 2005; Fambrini et al., 2006, 2011; Chapman et al., 2012). The double flowered mutant (*dbl*), with disc flowers displaying bilateral ray-like corollas, expresses *HaCYC2c* ectopically, an important loci for the establishment of ray flower identity. On the other hand, in the tubular-rayed (*tub*) mutants with ray flowers displaying tubular actinomorphic corollas (similar to the trumpet phenotype in *Anacyclus*; Figure 2B), *HaCYC2c* is expressed at lower levels due to the presence of transposable elements. Although we cannot suggest a direct ortholog gene of *HaCYC2c* in Anthemideae due its phylogenetic unstable position (Figure 1, Figure S1), qPCR analysis in *Anacyclus* suggests a lower expression of the *CYC2* genes *AcCYC2b, AcCYC2c* and *AcCYC2d* in the actinomorphic ray flowers of the trumpets (Figures 2B–D).

Our results support the role of CYC 2 genes in the evolution of Asteraceae flower morphological diversity and illustrate their evolution, diversification, and expression patterns in *Anacyclus*. From an evolutionary perspective, the phylogenetic analyses show that CYC2 gene family has diversified in Asteraceae into four main paralogs, which has been accompanied by an increased structural and functional complexity in inflorescences across the different lineages (Chapman et al., 2008; Tähtiharju et al., 2012). The comparison of gene expression analyses in CYC paralogs and their phylogenetic relationship suggests that different Asteraceae lineages have mostly conserved their roles in determining floral symmetry (Garcês et al., 2016). However, this work also confirms previous evidence proposed by Fambrini and Pugliesi (2017) for a consistent functional recruitment of CYC2 genes in the development of microspores (pollen) and macrospores (ovule) in female and bisexual flowers of the capitulum. This observation opens a new field for the study of the involvement of CYC2 genes in the evolution of sexual systems in Asteraceae.

## Author contributions

JF, MB, IA, and PC conceived the study. IA did the fieldwork, MB and IA maintained *Anacyclus* living collections, MB performed phylogenetic analyses, MB and PC conducted the *in situ* hybridization, MB and GS carried out the qPCR analysis, JF performed the speciation rate shifts analysis. MB, JF, IA, and PC discussed the results and wrote the manuscript.

### Conflict of interest statement

The authors declare that the research was conducted in the absence of any commercial or financial relationships that could be construed as a potential conflict of interest.

## Supplementary material

Figure S1 **Comparison of the CYC/TB1 summary trees based on nucleotide **(A,C,D)** and amino acid **(B)** data sets**.

Figure S2 **Maximum clade credibility (MCC) tree from the Bayesian Inference analysis of ***CYC/TB1***genes**.

Figure S3 **Longitudinal sections of floral tissues of ***A. clavatus*** hybridized with the sense probe**.

Figure S4 **Summary of expression patterns of selected CYC2 genes in the Asteraceae/Calyceraceae clade**.

Table S1 **CYC-like genes/clones isolated from ***Anacyclus*** and ***Matricaria*** (Asteraceae, Anthemideae)**.

Table S2 **Primers used for inverse PCR**.

Table S3 **Eudicot CYC-like genes included in the phylogenetic analyses**.

Table S4 **Datasets, trees and main statistics of the Bayesian analyses**.

Table S5 **Annealing temperature, product size and efficiency of the CYC2 gene specific primers used for qPCR in ***Anacyclus clavatus*** tissues**.

Table S6 **Main results of the qPCR essays performed on wild and trumpet individuals of ***A. clavatus*** following the Pfaffl method**.

Table S7 **Kolmogorov-Smirnoff test for probability distribution. α = 0.05**.

Table S8 **Ratio of expression (target EexpΔCt/ control EexpΔCt) of AcCYC2 genes in trumpet and wild individuals**.

Table S9 *****t***-test paired two sample for means of trumpet and wild expression of AcCYC2 genes. α = 0.05**.

Table S10 **Primers used to amplify the CYC2 gene specific probes for ***in situ*** hybridization in ***Anacyclus clavatus*****.

Table S11 **Main lineages reconstructed from the different Bayesian and ML analyses performed and their supports**.

Table S12 **Forty two ***CYC/TB1*** codons with significant evidence of positive selection according to the Mixed Effects Model of Episodic Selection (MEME) method**.
