# Supplementary figures and images for: Evolution and Expression Patterns of CYC/TB1 Genes in Anacyclus: Phylogenetic Insights for Floral Symmetry Genes in Asteraceae
Source: Front Plant Sci. 2017 Apr 25;8:589. doi: 10.3389/fpls.2017.00589 (PMC5403951; doi:10.3389/fpls.2017.00589)

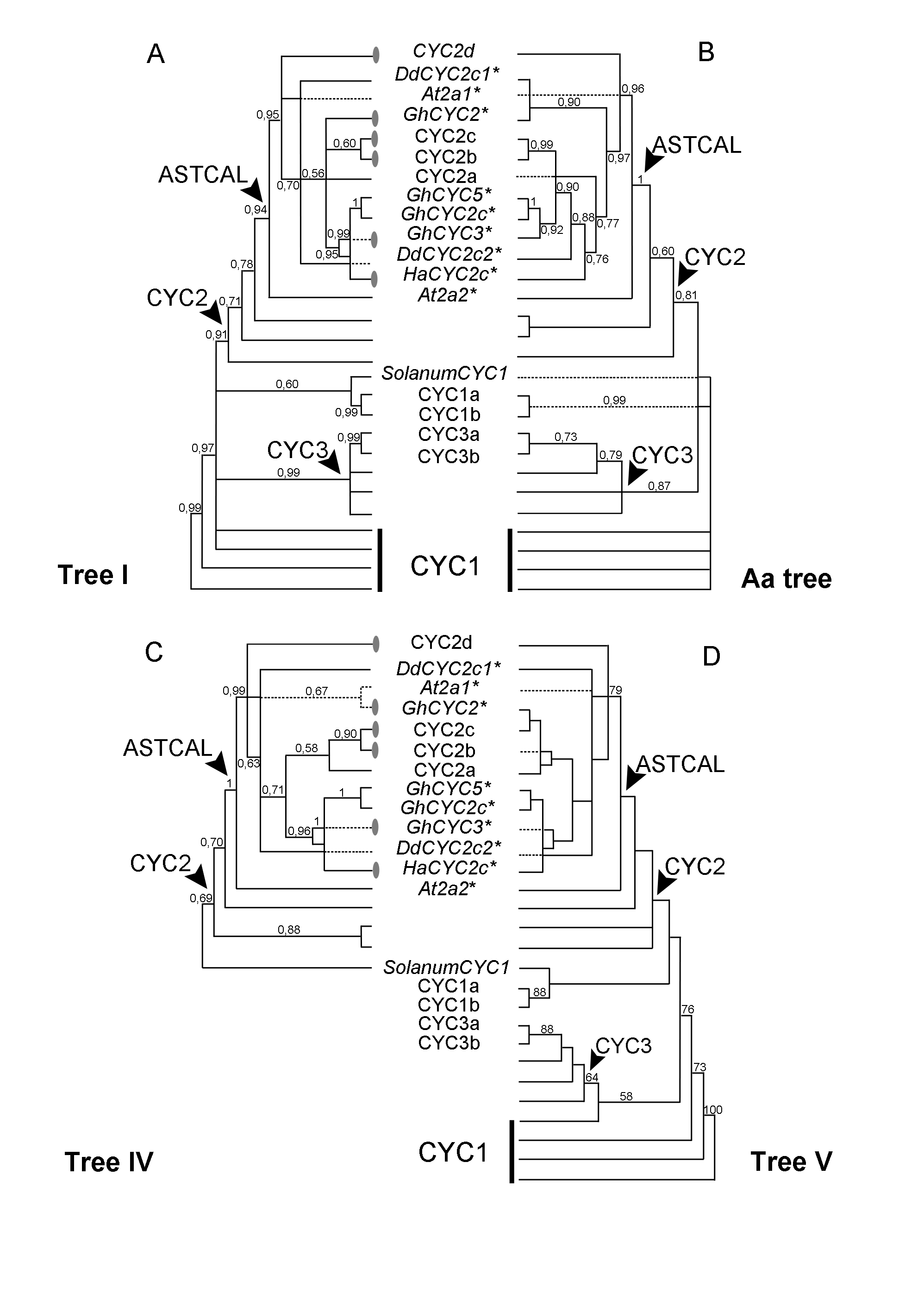

Supplement: Figure S1 — Comparison of the CYC/TB1 summary trees based on nucleotide (A,C,D) and amino acid (B) data sets. [file Image1.TIF]

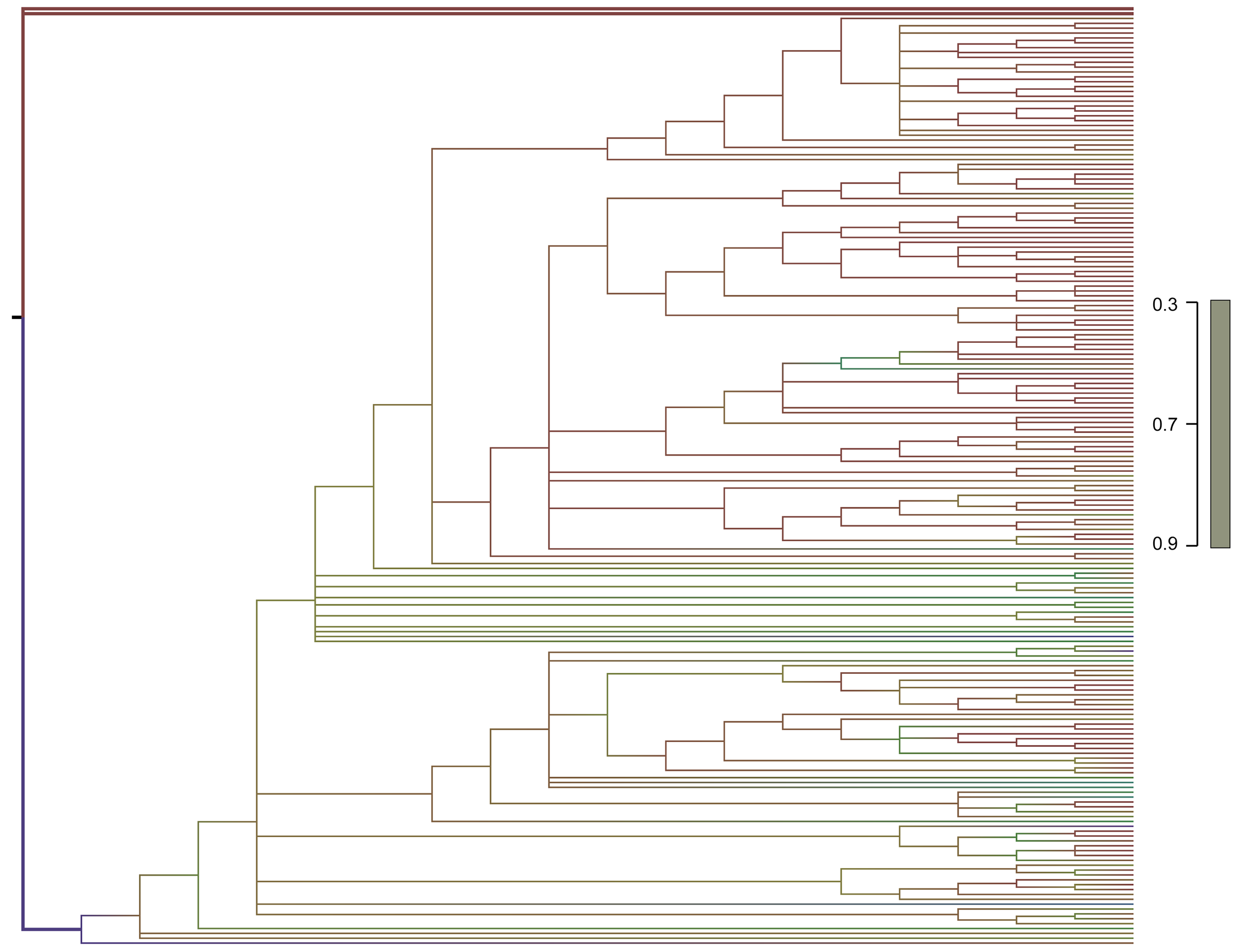

Supplement: Figure S2 — Maximum clade credibility (MCC) tree from the Bayesian Inference analysis of CYC/TB1genes. [file Image2.TIF]

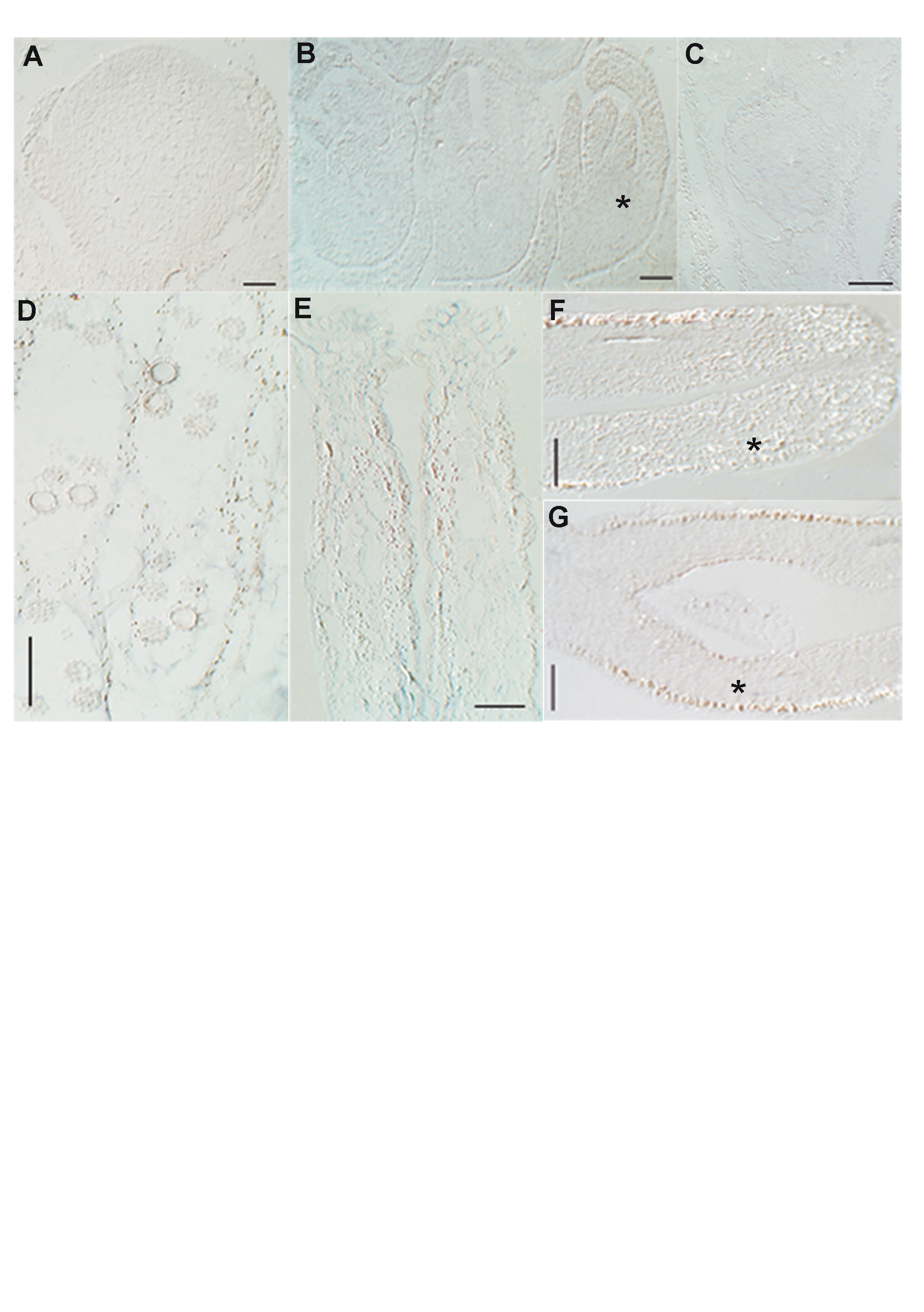

Supplement: Figure S3 — Longitudinal sections of floral tissues of A. clavatus hybridized with the sense probe. [file Image3.TIF]

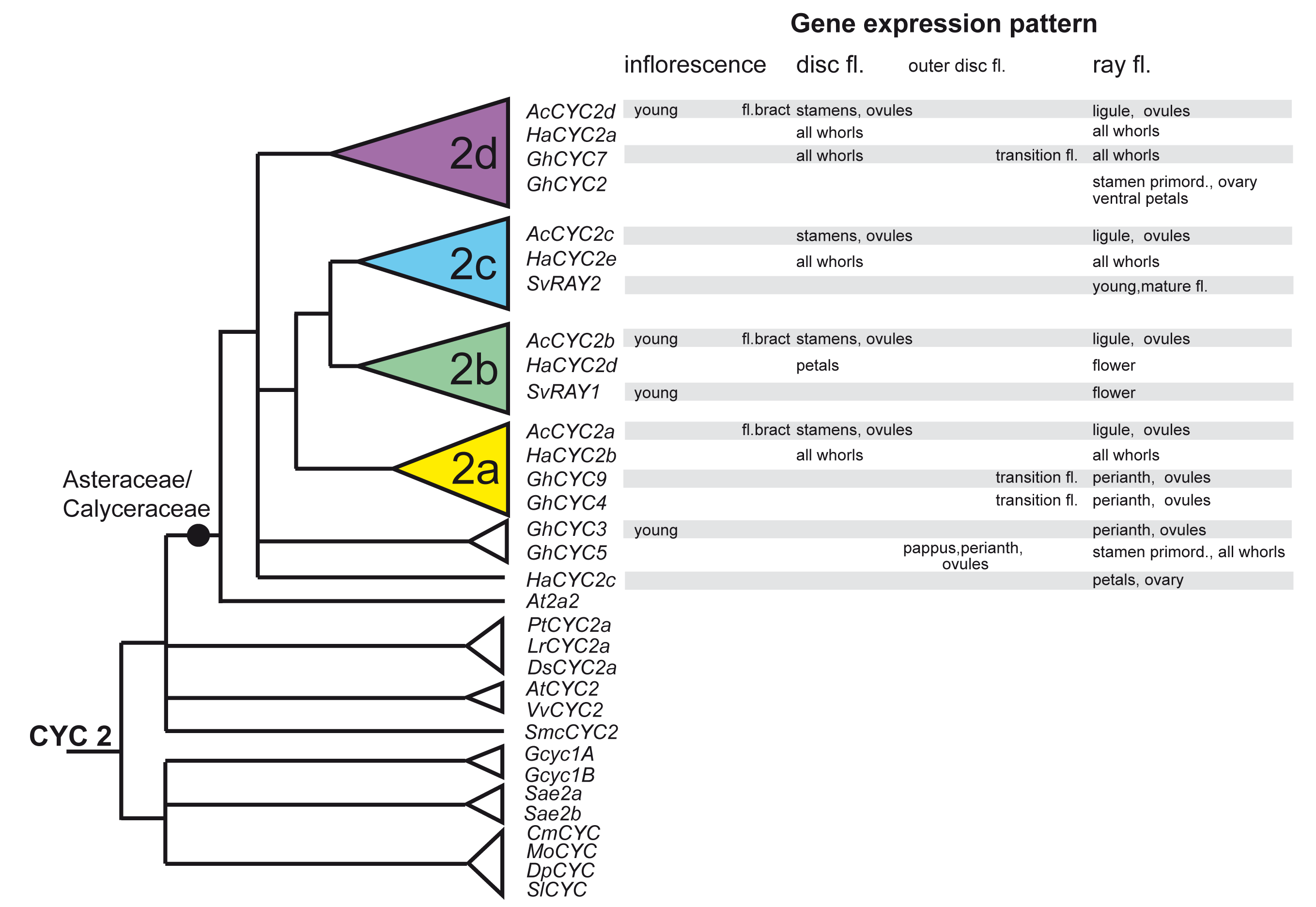

Supplement: Figure S4 — Summary of expression patterns of selected CYC2 genes in the Asteraceae/Calyceraceae clade. [file Image4.TIF]
